# A-G Score Associated With Outcomes in Solitary Hepatocellular Carcinoma Patients After Hepatectomy

**DOI:** 10.3389/fonc.2020.01286

**Published:** 2020-08-07

**Authors:** Guo Long, Junyi Shen, Ledu Zhou

**Affiliations:** ^1^Department of Liver Surgery, Xiangya Hospital, Central South University, Changsha, China; ^2^Department of Liver Surgery & Liver Transplantation Center, West China Hospital, Sichuan University, Chengdu, China

**Keywords:** alpha-fetoprotein, gamma-glutamyl transferase, HCC, hepatectomy, prognosis

## Abstract

**Aim:** The study aimed to investigate the clinical significance of preoperative alpha-fetoprotein (AFP) and gamma-glutamyl transferase (GGT) (A-G score) on hepatocellular carcinoma (HCC) patients.

**Methods:** A total of 474 solitary HCC patients were included. Survival analysis was evaluated by Kaplan-Meier method. Prognostic factors were analyzed in a multivariate model. The comparison of the predictive value of AFP, GGT, and A-G score was performed by receiver operating characteristic curve (ROC) analysis and decision curve analysis (DCA).

**Results:** Of the 474 patients, 137(28.9%), 241(50.8%), and 96(20.3%) patients were assigned to A-G score 0, 1, and 2, respectively. In multivariate analysis, A-G score, tumor size, microvascular invasion, tumor differentiation, satellite lesion, and state of HBV infection were independently predictive factors for RFS of solitary HCC patients. The A-G score could significantly stratify solitary HCC patients with a distinguished prognosis. The 1-, 3-, and 5-year RFS and OS among patients with A-G score 1 was better than that of patients with A-G score 2 and worse than that of patients with A-G score 0(all *p* < 0.05). Based on the result from the ROC analysis and DCA analysis, the A-G score appeared to be superior to either AFP or GGT alone in the prediction of prognosis of solitary HCC patients. In the subgroup analysis, the A-G score could accurately predict the prognosis of solitary HCC patients without MVI or with liver cirrhosis.

**Conclusions:** Preoperative A-G score could effectively and simply predict prognosis of solitary HCC patients after hepatectomy, especially for those with non-MVI solitary HCC or those with liver cirrhosis.

## Introduction

Hepatocellular cancer (HCC) has risen to become the fourth leading cause of cancer-related deaths worldwide, and its incidence rate continues to increase (1). China alone accounted for about 50% of HCC cases worldwide due to the high presence of hepatitis B virus (HBV) infection in the country, which led to a high mortality and considerable burden on the health service (2). Surgical resection and liver transplantation were potentially curative therapies for early stage HCC. Selected HCC patients receiving radical therapy could achieve up to a 70% of 5-year survival. However, about 80% of new HCC cases were diagnosed at an intermediate-advanced stage and had to receive palliative therapy, such as TACE or sorafenib. On the other hand, up to 70% of HCC patients might suffer from tumor relapse within 5 years after surgery, severely compromising the long-term survival of HCC patients. The overall survival of HCC patients was unsatisfactory. Factors affecting the prognosis need to be further elucidated. Barcelona Clinic Liver Cancer (BCLC) staging classification, proposed by Bruix et al., was widely adopted to stage HCCs and guide the management, and was also recommended by the AASLD (American Association for the Study of Liver Diseases) and EASL (European Association for the Study of the Liver) (3). For solitary HCCs, Bruix et al. classified single large HCC(>5 cm) as BCLC stage B (4). In contrast, Mazzaferro et al. firmly advocated for categorizing single HCCs as BCLC stage A, irrespective of tumor size (5). Till now, there had been no consensus about this issue. Actually, the controversy might be attributed to the fact that some patients with tumor size >5 cm among solitary HCC patients had a worse prognosis, lower than those within BCLC stage A (6). Furthermore, unfavorable prognostic factors, such as MVI and AFP, were commonly correlated with tumor size (7). Therefore, identifying the high-risk cohort of solitary HCC's preoperatively is of crucial importance to better staging solitary HCC and guiding comprehensive treatment.

Alpha-fetoprotein (AFP) is a useful parameter for the early detection of HCC. When developed, ~70% of HCC patients had elevated serum AFP. Recent studies suggested that high expression of AFP contributed to the malignancy of HCC cells, while the downregulation of AFP inhibited HCC cell proliferation and invasion (8). Clinically, preoperative serum AFP could be a prognostic indicator of prognosis following hepatectomy (9, 10). It also serves as an alarm to monitor tumor recurrence during follow-up and evaluate the outcome response to treatment for HCC (11, 12). Meanwhile, Hangzhou criteria takes AFP>400 ng/mL into account when selecting reliable and feasible candidates for liver transplantation with good outcomes (13). However, other investigators challenged this, believing that serum AFP might be not a good prognostic indicator for HCC patients (14). Some studies even considered that AFP had poor discriminatory power to predict the prognosis (15). In this situation, the combination of AFP with other tumor markers might improve the predictive ability of tumor recurrence and survival (16).

Serum gamma-glutamyl transferase (GGT) level was another biomarker associated with HCC (17). GGT as a membrane-bound glycoprotein plays a crucial role in the transfer of gamma-glutamyl residue from glutathione or related compounds, involved in nucleic acid metabolism and carcinogenesis. It was also a common indicator of hepatobiliary disease and liver cirrhosis (18). Clinically, the role of GGT may have been undervalued as it contributes little information to evaluate liver disease (vs. liver enzymes like ALT and AST). A recent study suggested that, in patients with viral hepatitis, serum GGT level positively correlated with HCC development, which might be a useful biomarker complementary to AFP for diagnosis of HCC (19). Furthermore, high GGT level correlated with poor clinicopathological features in HCC patients, including the degree of liver fibrosis, vascular invasion, and tumor size (17). Some studies reported that GGT was a promising prognostic predictor of HCC patients who underwent RFA (20) or TACE (21). In multivariate analysis, serum GGT and AFP levels used together was commonly considered as a risk prognostic factor in HCC patients. For instance, Li et al. found that GGT and AFP levels were independent risk factors for late recurrence (22). High levels of AFP and GGT was associated with macroscopic portal vein thrombosis (9). Moreover, GGT was also associated with prognosis in HCC with AFP ≤ 200 ng/mL (23). It indicated the GGT could further identify patients with poor prognosis even when they had normal serum AFP. Since GGT might be complementary to AFP for HCC diagnosis, the role of both markers in predicting the prognosis of HCC patients remains unclear. To our knowledge, the clinical significance of the combination of both markers for predicting postoperative prognosis has not been studied. It might also aid to identify high-risk groups of solitary HCC. Therefore, the current study aimed to clarify the predictive ability of the combination of AFP and GGT (A-G score) on solitary HCC patients after hepatectomy.

## Materials and Methods

### Patients

Patients who received hepatectomy for primary HCC between January 2014 and December 2015 in our hospital were extracted from a prospectively maintained database. All liver surgery was conducted by experienced hepatic surgeons. Variables including age, gender, status of hepatitis B virus (HBV) infection, liver cirrhosis, tumor diameter, satellite lesions, micro vascular invasion (MVI), and tumor differentiation were obtained. Preoperative routing blood tests, serum alanine aminotransferase (ALT), aspartate aminotransferase (AST), albumin (ALB), GGT, and AFP were also included. Microvascular invasion (MVI) was defined as the presence of tumor emboli within the central vein, the portal vein, or large capsular vessels (24). Lesions smaller than 2 cm and located within 2 cm of the main tumor were defined as satellite lesions (25). HCC was confirmed histologically by the experienced hepatic pathologists. Pathological information was extracted from the pathological reports. The inclusion criteria were as follows: (1) pathologically proven to be HCC, (2) single tumor, and (3) Child-Pugh class A or B patients. The exclusion criteria were as follows: (1) patients with other malignant tumors, (2) patients with positive surgical margin, (3) patients with lymph node metastasis, (4) patients with macrovascular invasion, (5) recurrent HCC, (6) patients re-treated by transplantation, and (7) uncomplete follow-up information. This study was approved by the Ethics Committee of Xiangya Hospital of Central South University, and informed consent was obtained from all HCC patients.

### Definitions

The serum AFP and GGT levels were simultaneously detected 2 days before operations. The cutoff value of AFP was 400 ng/mL, as reported in the literature. A score of 1 was assigned for patients with AFP>400 ng/mL. The serum GGT was dichotomized for RFS before log-rank test by using optimal cutoff values determined by the “*surviminer”* package of R software. A score of 1 was assigned for patients with GGT >43 U/L (Supplementary Figure 1). Based on the sum of the score of AFP or GGT, each patient could be classified into group A (A-G score 0), group B (A-G score 1), and group C (A-G score 2). The NLR was calculated by dividing neutrophil count by lymphocyte count. The PLR was estimated as platelet count divided by lymphocyte count. Major hepatectomy was defined as the removal of three segments or more, and minor hepatectomy was defined as the removal of ≤ 2 segments of the liver (26).

### Follow-Up

Antiviral therapy (entecavir or tenofovir) was recommended for HBV-related HCC patients according to the guidelines. After surgery, all HCC patients were followed up monthly for the first half-year, every 3 months for the first 2 years, and every 6 months in subsequent years. Blood test, liver function, tumor marker, and liver ultrasonography were completed at each visit. Suspected tumor recurrence in the liver or extrahepatic metastasis was confirmed by enhanced computed tomography (CT) and/or magnetic resonance imaging (MRI), or by biopsy. If recurrence was detected, patients received additional treatments. In order to optimize the management of recurrent HCC patients, a multidisciplinary approach (MDT) is recommended. The re-treatment options included surgical resection, radiofrequency ablation (RFA), transcatheter arterial chemoembolization (TACE), and best supportive care (BSC). Overall survival (OS) was defined as the time interval between the date of surgery to the date of death or last follow-up. Recurrence-free survival (RFS) was defined as the interval from surgery to recurrence or the date of the last follow-up.

### Statistical Analysis

Continual data was expressed as the mean ± standard deviation (M ± SD) and compared using Student's *t*-test. Continual data with non-parametric distribution was displayed as median and interquartile ranges (Q1-Q3) and compared by the non-parametric Mann-Whitney test. Categorical data was expressed as number and percentages, and compared by the *X*^2^ test, with the Yates correction, or Fisher's exact test. The cumulative RFS and OS rates were estimated by using the Kaplan-Meier method and compared using the log-rank test. A multivariable Cox regression model was performed to identify predictive factors for RFS or OS with forward and backward stepwise algorithms. Potential significant variables in univariable analysis (*p* < 0.1) was included in the Cox model. In this study, DCA and ROC analysis was used to compare the accuracy of predictive power on the prognosis among different variables. All statistical analysis was performed using R software (version 3.6.3, The R Foundation for Statistical Computing, Vienna, Austria). A *p* < 0.05 was considered statistically significant.

## Results

### Clinicopathological Characteristics

**Figure 1:**
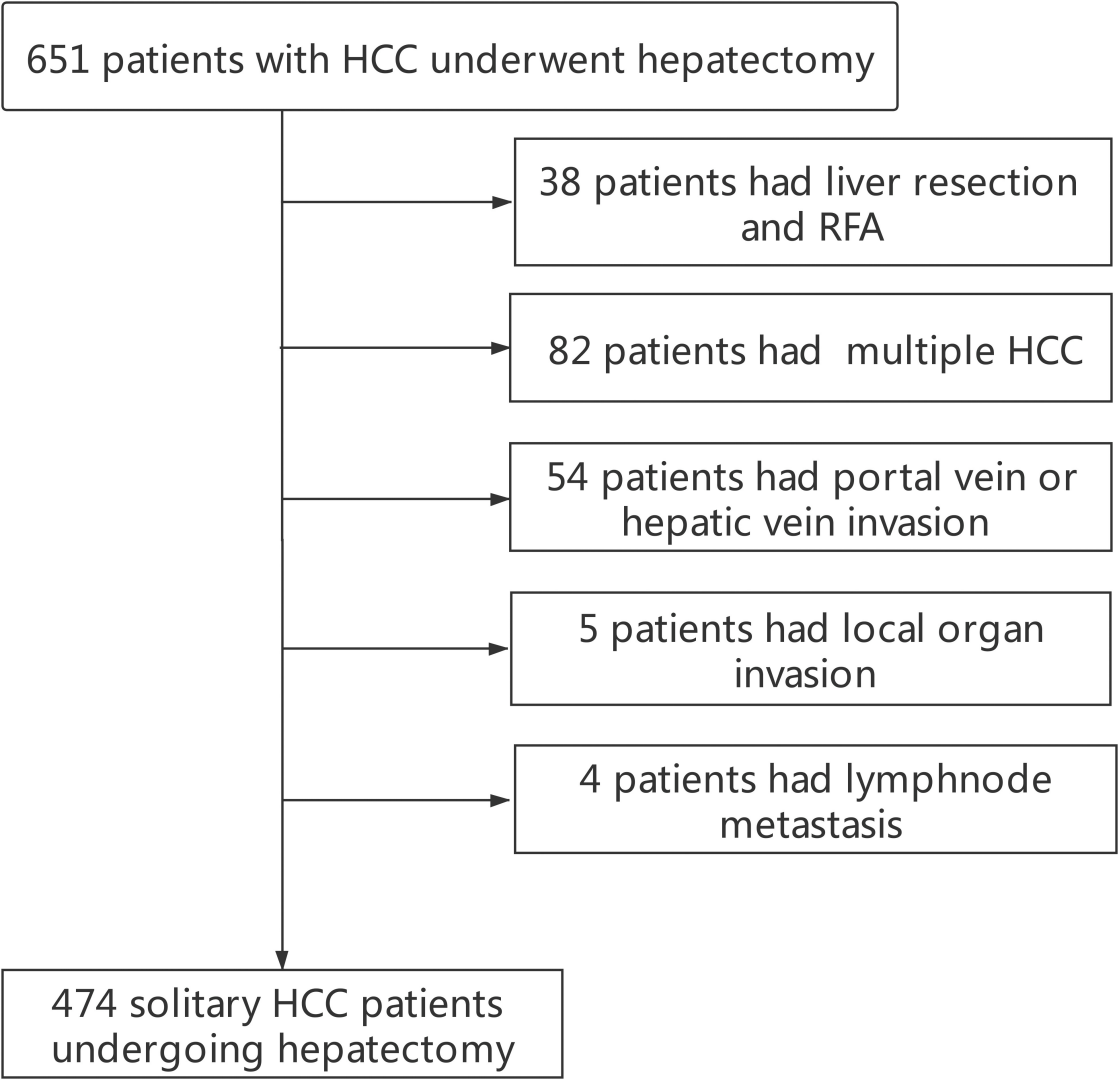
Flow chart about patients' selection. RFA, radiofrequency ablation.

Finally, a total of 474 solitary HCC patients were enrolled into this study (Figure 1). Demographics and clinicopathological features are displayed in Table 1. There were 392 men (82.7%) and 82 women (17.3%), with a median age of 49(12–82) years. 462 (97.5%) patients had positive HBcAb and 92(19.4%) patients had positive HBeAg. The presence of MVI and satellite lesions were observed in 124 (26.2%) patients and 45 (9.5%) patients, respectively. Liver cirrhosis was observed in 291 (61.4%) cases. Tumor differentiation was moderate-well in 289 (61.0%) cases and poor in 185(39.0%) cases. The tumor diameter in the whole cohort was 6.1 ± 3.6 cm. 140 (29.5%) patients had AFP ≤ 400 ng/mL. Since the serum GGT level displayed non-normal distribution, the optimal cutoff value of GGT groups was identified as 43 U/L for RFS with *Maximally Selected Rank Statistics*. Finally, 293 (61.8%) patients had GGT > 43 U/L. Based on the results from the combination of AFP and GGT, the enrolled patients were divided into three groups with group A [A-G score 0, 134(28.9%)], group B [A-G score 1, 241(50.8%)], and group C [A-G score 2, 96(20.3%)] (Supplementary Figure 2).

**Table 1:** Clinicopathological characteristics grouped by the A-G score.

		**group A**	**group B**	**group C**	***p*-value**
Variables	*n* = 474	*n* = 137	*n* = 241	*n* = 96	
Age (y)	49 (42–59)	51 (44–61)	50 (43–59)	45 (39–55)	<0.001
Gender					0.395
Female	82 (17.3)	21 (15.3)	40 (16.6)	21 (21.9)	
Male	392 (82.7)	116 (84.7)	201 (83.4)	75 (78.1)	
Positive HBsAg	412 (86.9)	118 (86.1)	210 (87.1)	84 (87.5)	0.945
Positive HBeAg	92 (19.4)	22 (16.1)	42 (17.4)	28 (29.2)	0.024
Positive HBcAb	462 (97.5)	135 (98.5)	234 (97.1)	93 (96.9)	0.634
Liver cirrhosis	291 (61.4)	83 (60.6)	155 (64.3)	53 (55.2)	0.293
MVI	124 (26.2)	25 (18.2)	57 (23.7)	42 (43.8)	<0.001
Satellite lesion	45 (9.5)	4 (2.9)	31 (12.9)	10 (10.4)	0.006
Differentiation					0.003
Moderate-Well	289 (61.0)	89 (65.0)	156 (64.7)	44 (45.8)	
Poor	185 (39.0)	48 (35.0)	85 (35.3)	52 (54.2)	
Tumor diameter (cm)	6.1 (3.6)	4.2 (1.9)	5.9 (3.5)	9.1 (3.8)	<0.001
Plate (10^9^/L)	146.6 (69.9)	136.0 (54.6)	142.7 (70.6)	171.5 (81.4)	<0.001
Neutrophil (10^9^/L)	3.45 (1.40)	3.20 (1.38)	3.47 (1.40)	3.77 (1.38)	0.009
Lymphocyte (10^9^/L)	1.53 (0.57)	1.51 (0.53)	1.55 (0.57)	1.52 (0.63)	0.808
Macrophage (10^9^/L)	0.37 (0.16)	0.34 (0.15)	0.38 (0.17)	0.39 (0.16)	0.015
INR	1.1 (0.1)	1.1 (0.1)	1.1 (0.1)	1.1 (0.1)	0.795
Fib	2.8 (1.1)	2.5 (0.7)	2.9 (1.2)	3.1(1.0)	<0.001
TBIL (μmol/L)	14.9 (6.5)	14.1(5.3)	15.2 (7.3)	15.5 (5.8)	0.149
ALT (U/L)	38.5 (27–59)	30 (22–43)	43 (30–65)	41.5 (28.7–59.0)	<0.001
AST (U/L)	37 (28–53.75)	28 (24–36)	39 (31–55)	46.5 (33–74)	<0.001
ALB (g/L)	41.3 (4.3)	41.9 (4.3)	41.1 (4.3)	40.8 (4.3)	0.146
GGT (U/L)	56 (31.3–108.7)	27 (18–36)	74 (48–133)	92.5 (64.7-160.5)	<0.001
CREA	74.6 (16.2)	76.8 (13.8)	74.8 (16.3)	71.1 (18.5)	0.027
NLR	2.5 (1.6)	2.4 (1.7)	2.5 (1.4)	2.9 (1.8)	0.043
PLR	104.1 (57.0)	96.2 (41.7)	99.8 (54.6)	125.8 (74.3)	<0.001
AFP (ng/mL)					<0.001
>400	334 (70.5)	137 (100.0)	197 (81.7)	0 (0.0)	
≤ 400	140 (29.5)	0 (0.0)	44 (18.3)	96 (100.0)	
GGT (U/L)					<0.001
≤ 43	181 (38.2)	137 (100.0)	44 (18.3)	0 (0.0)	
>43	293 (61.8)	0 (0.0)	197 (81.7)	96 (100.0)	
A-G score					<0.001
0	137 (28.9)	137 (100.0)	0 (0.0)	0 (0.0)	
1	241 (50.8)	0 (0.0)	241 (100.0)	0 (0.0)	
2	96 (20.3)	0 (0.0)	0 (0.0)	96 (100.0)	
Hepatectomy					0.005
Minor	241 (50.8)	78 (56.9)	128 (53.1)	35 (36.4)	
Major	233 (49.2)	59 (43.1)	113 (46.9)	61 (63.6)	
Recurrence					0.004
Within BCLC A	99 (34.6)	33 (50.8)	48 (32.2)	18 (25.0)	
Beyond BCLC A	187 (65.4)	32 (49.2)	101 (67.8)	54 (75.0)	
Re-treatment					0.001
BSC	66 (23.1)	12 (18.5)	39 (26.2)	15 (20.8)	
TACE	116 (40.6)	18 (27.7)	55 (36.9)	43 (59.7)	
RFA	44 (15.4)	16 (24.6)	22 (14.8)	6 (8.3)	
Resection	60 (21.0)	19 (29.2)	33 (22.1)	8 (11.1)	

*HBsAg, hepatitis B surface antigen; HBeAg, hepatitis B 'e' antigen; HBcAb, hepatitis B core antibody; MVI, microvascular invasion; TBIL, total bilirubin; ALT, Alanine aminotransferase; AST, aminotransferase; ALB, albumin; GGT, γ-glutamyl transpeptidase; NLR, Neutrophil-to-Lymphocyte Ratio; PLR, Platelet-to-lymphocyte ratio; AFP, alpha-fetoprotein; BCLC, Barcelona Clinic Liver Cancer; BSC, best supportive care; RFA, radiofrequency ablation*.

With regard to the status of HBV infection, there was no difference in the HBsAg and HBcAb among the three groups. Notably, group C had a higher rate of patients with positive HBeAg, suggesting a more active HBV infection. In term of features of tumor invasion, compared with group A or B, group C had significantly larger tumor diameters, a higher rate of MVI and satellite lesions, and more patients with poor differentiation. There was no significant difference in the rate of liver cirrhosis. Regarding systemic inflammation, group C had higher NLR levels and PLR levels, indicating higher levels of systemic inflammation. Lymphocyte count among the three groups was similar. As to liver function, group B and group C had higher serum TBIL, ALT, and AST than that of group A. However, there was no significant difference in serum ALB or INR among the three groups.

In patients with recurrent HCC, the proportion of patients who developed BCLC A stage recurrent HCC in group A was 50.8% (group B:32.2%; group C:25.0%). Moreover, up to 53.8% of patients in group A received surgical resection or RFA, while in group B it was 36.9% and for group C was 19.4%.

### Higher A-G Score Associated With Worse Prognosis

As shown in Table 2, the results of the univariate analysis of prognostic factors for RFS suggested there were twelves potential prognostic factors. In multivariate analysis, tumor diameter (HR:1.07, 95%CI:1.03–1.11, *p* < 0.001), MVI (HR:1.61, 95%CI:1.23–2.11, *p* < 0.001), satellite lesions (HR:2.47, 95%CI:1.74–3.50, *p* < 0.001), tumor differentiation (HR:1.37, 95%CI:1.08–1.74, *p* = 0.009), HBV (HR:1.49, 95%CI:1.00–2.22, *p* = 0.049), HBeAg (HR:1.59, 95%CI:1.2032.10, *p* = 0.001), and high A-G score (HR:1.23, 95%CI:1.01–1.49, *p* = 0.037) remained as independent predictors of RFS. Similarly, the results of the Cox regression hazard model to determine prognostic risk factors for OS were shown in Table 3; a total of 10 potential prognostic factors entered into the Cox model. Finally, the six variables retained in the Cox model were: tumor diameter (HR:1.11, 95%CI:1.1.07–1.15, *p* < 0.001), MVI (HR:1.58, 95%CI:1.17–2.13, *p* = 0.002), satellite lesions (HR:2.62, 95%CI:1.78–3.87, *p* < 0.001), tumor differentiation (HR:1.51, 95%CI:1.13–2.00, *p* = 0.004), HBeAg (HR:1.89, 95%CI:1.38–2.60, *p* < 0.001), and high A-G score (HR:1.18, 95%CI:0.93–1.48, *p* = 0.157).

**Table 2:** Univariate and multivariate analysis of prognostic factors contributing to RFS after hepatectomy.

**Variable**	**HR**	**95%CI**	***p*-value**	**HR**	**95%CI**	***p*-value**
Age	0.98	(0.97–0.99)	0.026			
Gender	1.16	(0.84–1.61)	0.341			
HBV	1.62	(1.10–2.40)	0.014	1.49	(1.00–2.22)	0.049
HBeAg	1.73	(1.32–2.26)	<0.001	1.59	(1.20–2.10)	0.001
HBcAb	1.35	(0.60–3.04)	0.461			
Liver cirrhosis	0.89	(0.70–1.13)	0.379			
Tumor diameter	1.10	(1.07–1.13)	<0.001	1.07	(1.03–1.11)	<0.001
MVI	2.09	(1.63–2.68)	<0.001	1.61	(1.23–2.11)	<0.001
Satellite lesion	2.46	(1.74–3.47)	<0.001	2.47	(1.74–3.50)	<0.001
Differentiation	1.51	(1.19–1.91)	<0.001	1.37	(1.08–1.74)	0.009
TBIL	1.00	(0.98–1.02)	0.584			
ALT	1.00	(0.99–1.00)	0.435			
AST	1.00	(1.00–1.00)	0.004			
ALB	0.99	(0.96–1.01)	0.598			
Fib	1.09	(0.98–1.20)	0.090			
INR	1.34	(0.47–3.86)	0.578			
M	1.71	(0.87–3.35)	0.118			
PLR	1.00	(1.00–1.00)	0.028			
NLR	1.04	(0.97–1.11)	0.236			
CREA	1.00	(0.99–1.00)	0.945			
A-G score	1.62	(1.37–1.92)	<0.001	1.23	(1.01–1.49)	0.037

**Table 3:** Univariate and multivariate analysis of factors associated with OS after hepatectomy.

**Variables**	**HR**	**95%CI**	***p*-value**	**HR**	**95%CI**	***p*-value**
Age	0.99	(0.98–1.00)	0.383			
Gender	1.13	(0.77–1.65)	0.506			
HBV	1.24	(0.80–1.91)	0.331			
HBeAg	1.86	(1.36–2.54)	<0.001	1.89	(1.38–2.60)	<0.001
HBcAb	1.05	(0.43–2.56)	0.902			
Liver cirrhosis	0.78	(0.59–1.03)	0.087			
Tumor diameter	1.12	(1.09–1.16)	<0.001	1.11	(1.07–1.15)	<0.001
MVI	2.16	(1.62–2.87)	<0.001	1.58	(1.17–2.13)	0.002
Satellite lesion	2.43	(1.65–3.56)	<0.001	2.62	(1.78–3.87)	<0.001
Differentiation	1.65	(1.25–2.17)	<0.001	1.51	(1.13–2.00)	0.004
TBIL	1.00	(0.98–1.02)	0.739			
ALT	0.99	(0.99–1.00)	0.807			
AST	1.00	(1.00–1.00)	0.001			
ALB	0.97	(0.94–1.00)	0.078			
INR	1.08	(0.29–3.98)	0.899			
Fib	1.22	(1.10–1.36)	<0.001			
NLR	1.07	(1.00–1.15)	0.039			
PLR	1.00	(1.00–1.00)	<0.001			
CREA	0.99	(0.98–1.00)	0.334			
A-G score	1.72	(1.41–2.11)	<0.001	1.18	(0.93–1.48)	0.157

*HR, hazard ratio; CI, confidence interval*.

**Figure 2:**
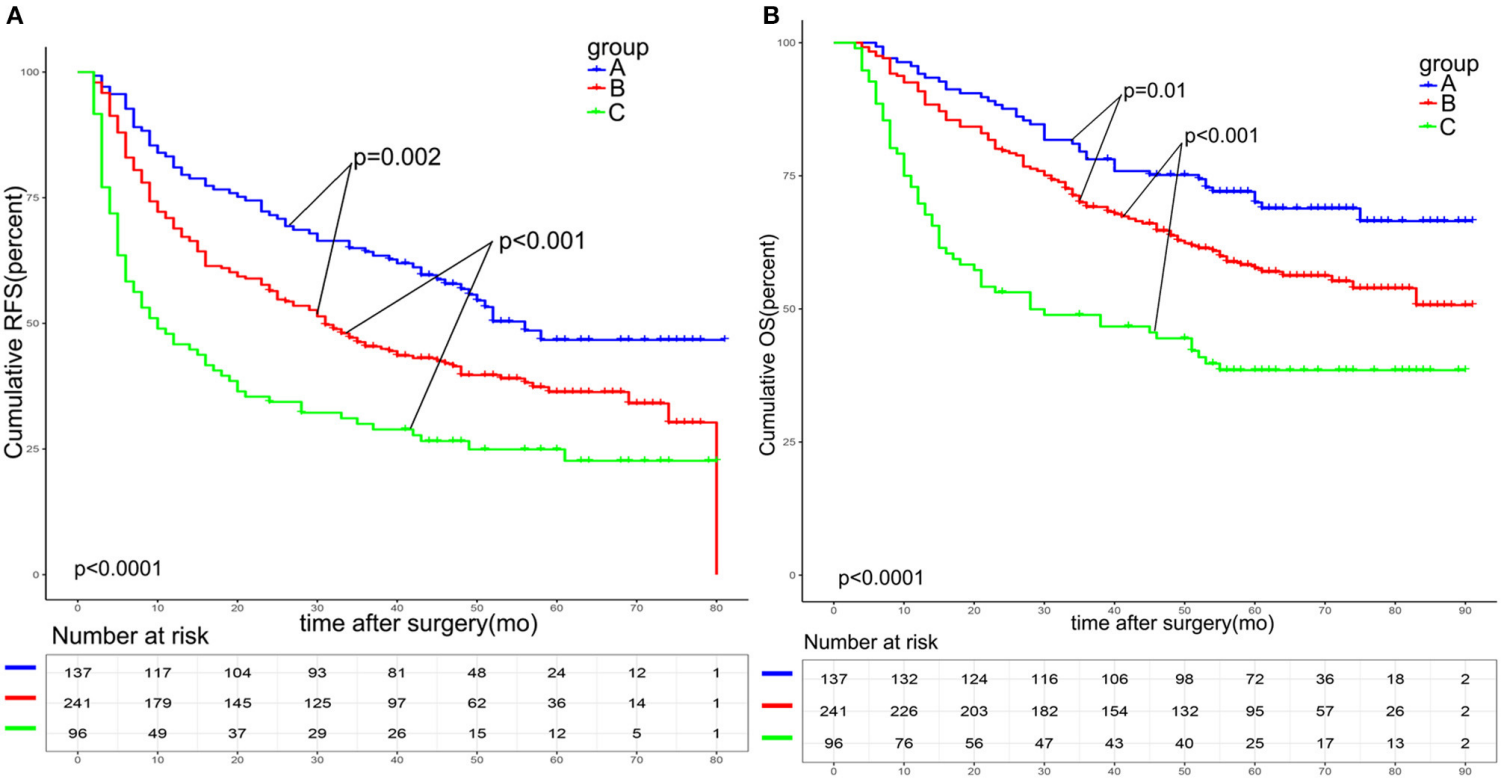
Survival analysis based on the A-G score. The 1-, 3-, and 5-year RFS **(A)** and OS **(B)** among patients with an A-G score of 1 was better than that of patients with an A-G score of 2 and worse than that of patients with an A-G score of 0(all *p* < 0.05). RFS: recurrence free survival. OS: overall survival.

As shown in Figure 2, the 1-, 3-, and 5-year RFS and OS rates in group A were 81.0, 64.2, and 46.7%, and 94.2, 78.1, and 70.0%, respectively. The 1-, 3-, and 5-year RFS and OS rates in group B were 68.9, 45.4, and 36.3%, and 90.9, 69.2, and 57.6%, respectively. The 1-, 3-, and 5-year RFS and OS rates in group C were 45.8, 30.0, and 24.9%, and 69.8, 48.9, and 38,5%, respectively. These results indicated that group B had a significantly better prognosis than that of group C (RFS: *p* < 0.001; OS: *p* = 0.001), but worse than that of group A (RFS: *p* < 0.001; OS: *p* = 0.01). Higher A-G score was associated with worse prognosis.

### Comparative of Predictive Ability of A-G Score, AFP and GGT

**Figure 3:**
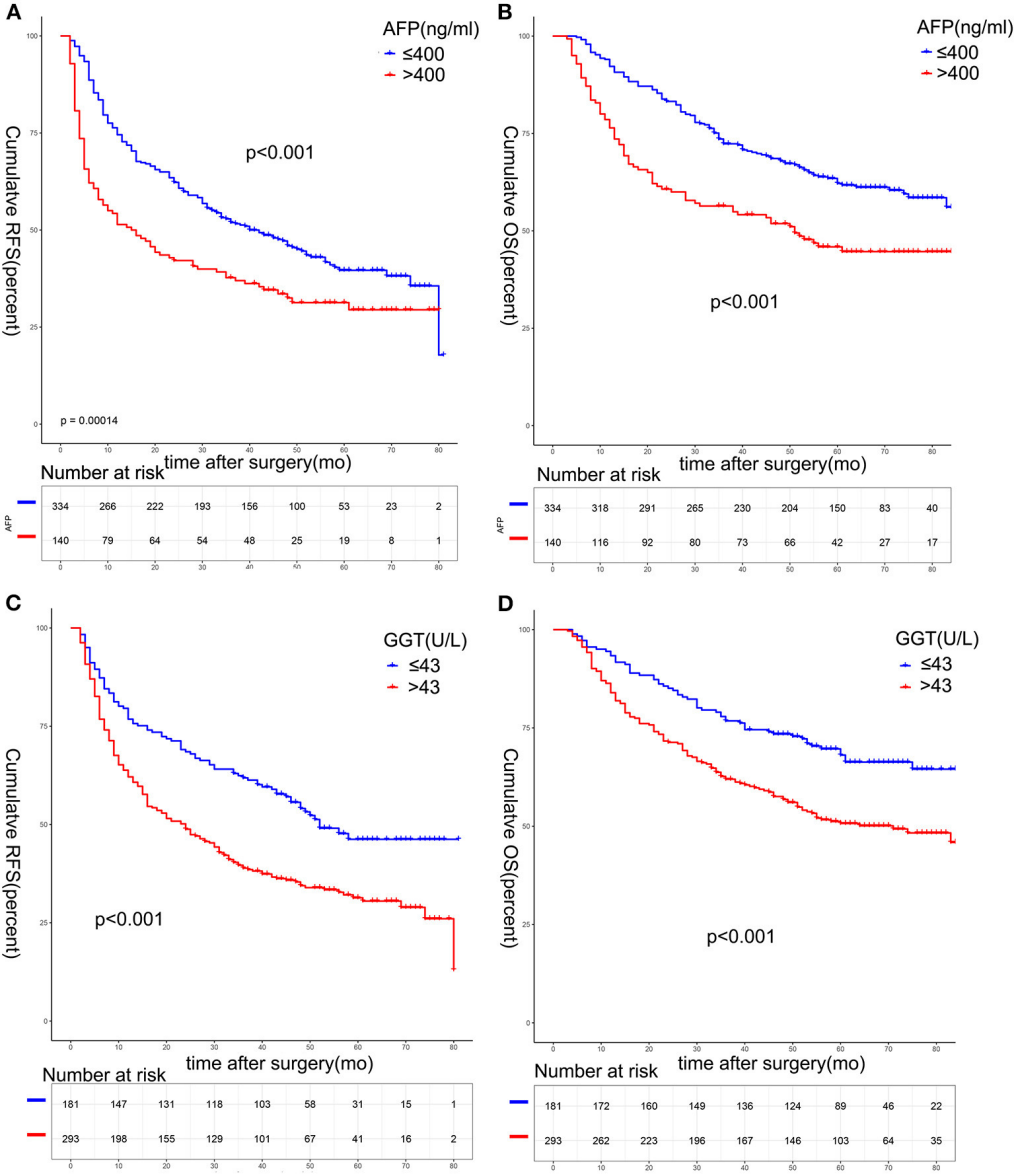
Survival analysis based on serum AFP or GGT. The 1-, 3-, and 5-year RFS **(A)** and OS **(B)** among patients with AFP ≤ 400 ng/mL was better than that of patients with AFP > 400 ng/mL (all *p* < 0.05). The 1-, 3-, and 5-year RFS **(C)** and OS **(D)** among patients with GGT ≤ 43 U/L was better than that of patients with GGT > 43 U/L (all *p* < 0.05).AFP, alpha-fetoprotein.

There was statistical difference in RFS and OS between the two groups divided according to serum AFP level among solitary HCC patients (>400 vs. ≤ 400 ng/mL) (Figures 3A,B). The 1, 3, and 5-year RFS rates were 74.6, 51.9, and 39.6% in patients with AFP ≤ 400 ng/mL, and 51.4, 37.7, and 31.3% in patients with AFP >400 ng/mL, respectively (*p* < 0.001). The 1, 3, and 5-year OS rates were 92.2, 72.3, and 62.2% in patients with AFP ≤ 400 ng/mL, and 76.4, 56.4, and 45.9% in patients with AFP > 400 ng/mL, respectively (*p* < 0.001). When patients were stratified by the serum GGT level (>43 vs. ≤ 43 U/L), patients with higher serum GGT levels had worse RFS and OS rates (Figures 3C,D). The 1, 3, and 5-year RFS rates were 76.8, 61.9, and 46.2% in patients with GGT ≤ 43 U/L, and 62.1, 38.9, and 30.5% in patients with GGT > 43 U/L (*p* < 0.001). The 1, 3, and 5-year OS rates were 93.4, 76.8, and 68.1% in patients with ≤ 43 U/L, and 84.0, 62.0. and 50.7% in patients with GGT > 43 U/L, respectively (*p* < 0.001).

**Figure 4:**
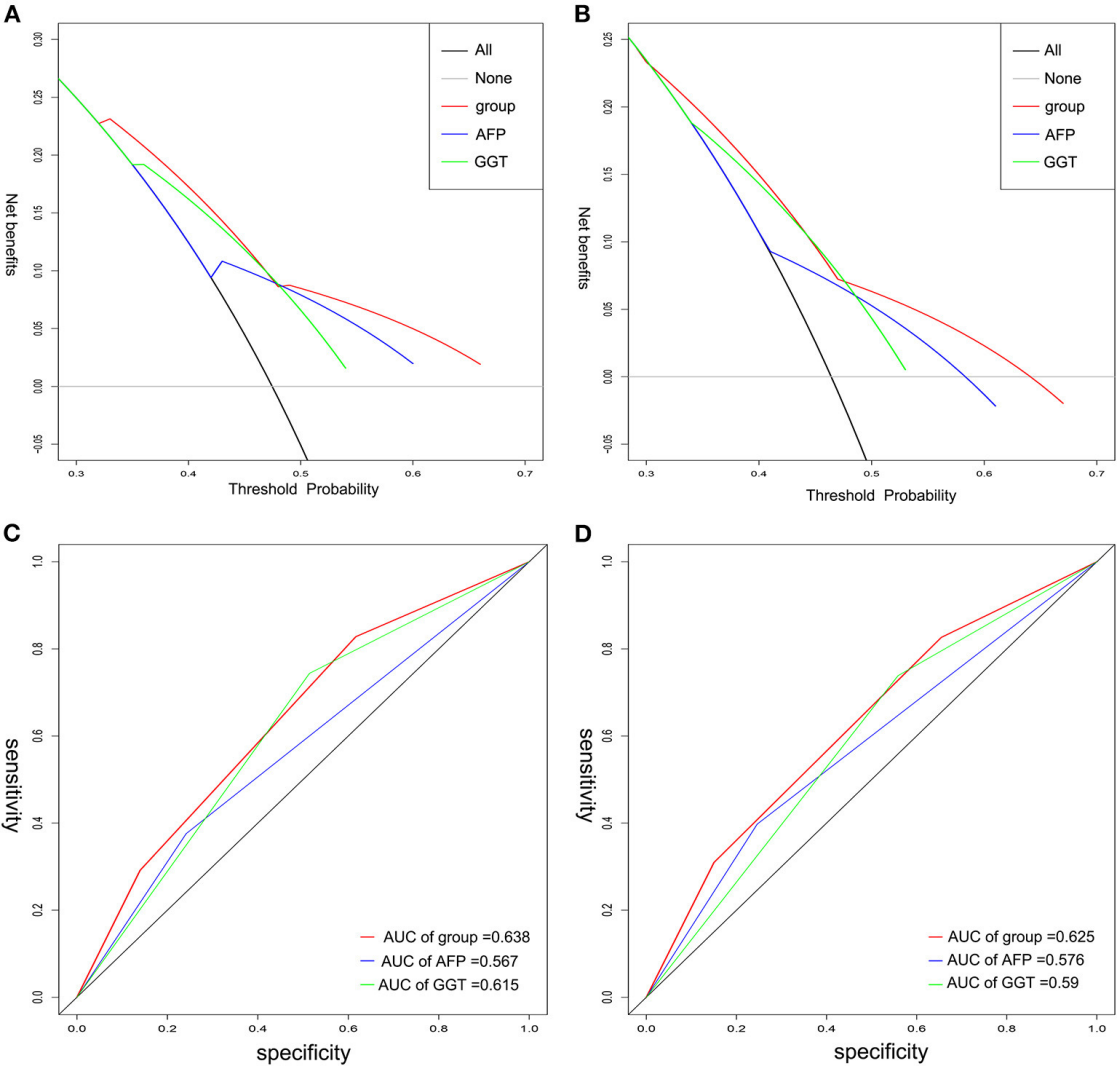
The time-dependent ROC and DCA curves of A-G score, AFP, and GGT on 3-year RFS and OS. Decision curve analysis for RFS **(A)** and OS **(B)** indicated the A-G score was better. The AUC of A-G score on RFS **(C)** and OS **(D)** was higher than that of either AFP or GGT. ROC, receiver operating characteristic; DCA, decision curve analysis.

We performed decision curve analysis (DCA) for evaluation of the predictive value of the tumor marker (Figures 4A,B). DCA demonstrated that the A-G score showed a better net benefit for 3-year RFS and OS than that of AFP or GGT alone, suggesting a higher predictive accuracy. Furthermore, receiver operating characteristic (ROC) curves was used to compare its accuracy in predicting the prognosis (Figures 4C,D). The AUC of A-G score in predicting 3-year RFS was 0.638 (AFP: 0.567; GGT: 0.615) and the AUC of A-G score for predicting 3-year OS was 0.625 (AFP: 0.576; GGT: 0.590), which suggested the A-G score might be superior to the AFP or GGT alone.

### Predictive Value of A-G Score in the Subgroup Analysis

**Figure 5:**
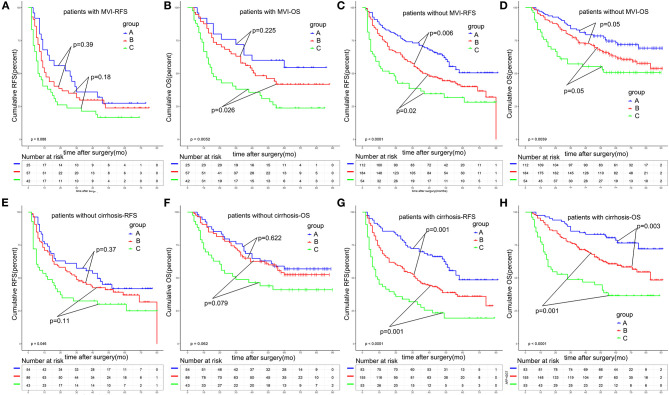
Subgroup survival analysis based on MVI or liver cirrhosis. The A-G score could significantly stratify the prognosis of solitary HCC patients without MVI **(A–D)** and patients with liver cirrhosis **(E–H)**. MVI, microvascular invasion.

Microvascular invasion (MVI) has been widely reported to be a poor prognostic factor for HCC. Patients with the presence of MVI were at a high risk of recurrence. In order to validate the predictive value of A-G score, we made a subgroup analysis based on whether patients displayed the presence of MVI. As shown in Figures 5A–D, the A-G score performed well in stratifying the patients with distinguished prognosis in patients with MVI or without MVI. Particularly, for patients without MVI, the 1, 3, and 5-year RFS rates were 74.6, 51.9, and 39.6% in patients of group A, and 51.4, 37.7, and 31.3% in patients of group C, respectively (*p* < 0.001). The 1, 3, and 5-year OS rates were 75.9, 57.3, and 50.8% in patients in group C, 92.9, 73.2, and 62.5% in group B, and 95.5, 81.3, and 73.4% in group A, respectively (*p* = 0.003). There was statistical significance among the three groups.

Furthermore, we made a subgroup analysis based on whether HCC patients developed liver cirrhosis (Figures 5E–H).We found that, in patients without liver cirrhosis, the A-G score had a poor ability to predict the different prognosis of HCC patients, with the *p*-value of RFS and OS being 0.046 and 0.062. However, for patients with liver cirrhosis, the 1, 3, and 5-year RFS rates were 43.4, 27.2, and 19.5% in patients of group C, and 51.4, 37.7, and 31.3% in patients of group A, respectively (*p* < 0.001). The 1, 3, and 5-year OS rates were 68.4, 45.5, and 36.0% in patients in group C, 85.5, 68.7, and 48.6% in patients group B, and 96.4, 84.3, and 78.6% in patients group A, respectively (*p* < 0.001). A-G score could significantly predict the prognosis of solitary HCC patients with liver cirrhosis.

## Discussion

Solitary HCC is a subgroup of HCC patients with high heterogeneity. Some investigators believed solitary HCC patients could achieve comparable outcomes as HCC patients within BCLC stage A. However, numerous studies indicated that increased tumor size correlated with a high presence of unfavorable pathological features, such as MVI and satellite lesions, which greatly impaired the prognosis of solitary HCC patients. Identifying high-risk groups of solitary HCC was necessary. On the other hand, understanding the role of tumor markers in HCC was of considerable significance for clinical practice. Since there was less of an impact from some major risk factors, such as tumor number and major vascular invasion, it was easy to investigate the clinical value of tumor markers. For the above reason, we selected solitary HCC patients in our current study.

Tumor markers had been widely used in clinical practice. Some studies indicated that serum AFP or GGT played decisive roles in the diagnosis of HCC or prediction of prognosis of HCC patients after surgery (20, 27, 28). High serum AFP or GGT was robustly linked to unfavorable clinicopathological features (15, 21). However, the optimal cutoff value of serum AFP or GGT in the prediction of HCC prognosis varied (15, 23, 28). Since the serum AFP of some patients in the current study had no concrete value when beyond the upper limit, as previous studies of other large-volume and high-quality liver surgery center described, the cutoff value of serum AFP was defined as 400 ng/mL (13, 15). In order to better define the role of GGT in the prediction of HCC' prognosis, serum GGT level as a continuous variable was dichotomized for RFS. We introduced a method for determining the optimal pointcut of GGT based on the statistics of maxstat (ranking statistics of the largest selection), performed by the *survminer* package of R software. Finally, the optimal cutoff value was defined as 43 U/L.

The role of AFP on the prognosis remains controversial (14, 15). Some researchers considered that AFP could predict the prognosis of HCC patients, while some other investigators did not. A possible reason for this disagreement might be the varied cutoff value, specific subgroup of HCC, or the answer being concealed by some other major effectors, such as multiple tumors and major vascular invasion, which leads to a controversial role of AFP in HCC. In our current study, we confirmed that patients with AFP > 400 ng/mL were predicted the worst prognosis of solitary HCC patients. Several studies have shown the negative impact of GGT on the prognosis of HCC with a cutoff value of 40, 50, 75, or 88 U/L (20, 21, 23, 28). Using an outcome-oriented method (maximally selected rank statistics), we found that solitary HCC patients with GGT > 43 U/L had a worse outcome. In terms of the relationship between serum AFP and GGT, some studies showed that the serum GGT positively correlated with serum AFP levels (21). Some studies have indicated that serum GGT was not associated with serum AFP level (20, 23, 28). Another study indicated that both markers were complementary to each other (28). In the current study, we found that the majority of HCC patients had AFP > 400 ng/mL (70.5%) or GGT > 43 U/L (61.8%). Based on our classification, all patients could be classified into three groups. Patients with AFP > 400 ng/mL and GGT > 43 U/L accounted for 20.3%. Patients with AFP ≤ 400 ng/mL and GGT ≤ 43 U/L shared 28.9% of the total patients. There were 50.8% of solitary HCC patients who had either AFP > 400 ng/mL or GGT > 43 U/L. To expand these findings, we assessed whether the combination of AFP and GGT could better predict the prognosis of solitary HCC patients.

Interestingly, the prognosis in term of RFS and OS among patients with an A-G score of 1 was better than that of patients with an A-G score of 2 and worse than that of patients with an A-G score of 0. The A-G score could effectively and simply stratify three subgroups of solitary HCC patients with distinguished prognosis. These results suggest that a higher A-G score was negatively associated with worse outcomes. When stratified by serum AFP or GGT, only two different groups could be identified. Moreover, the ROC analysis and DCA analysis indicated that the A-G score appeared to be superior to either AFP or GGT in the prediction of the prognosis of solitary HCC patients. In the Cox model, preoperative A-G score, but not serum AFP or GGT, was an independent prognostic risk factor associated with RFS(HR:1.23). Although it was not statistically significantly related to OS, it was still retained in the Cox model. Since the factors related to OS were more complex when recurrence status and treatment were incorporated, we considered its role in RFS could better represent its significance in HCC. Therefore, A-G score had a definite predictive role in HCC. An A-G score of 0 represented a low-risk cohort of solitary HCC patients while an A-G score of 2 highly suggested a group of solitary HCC patients at high risk of recurrence and poor outcome. This A-G score could offer an objective tool to guide clinical decision-making in solitary HCC patients.

MVI has been widely investigated to be a poor prognostic factor of RFS and OS for HCC (29). In keeping with this, MVI was identified as a risk factor associated with prognosis of solitary HCC patients. To date, the presence of MVI had caught enough attention from hepatic surgeons due to it strongly indicating that HCC patients tended to suffer from HCC recurrence and poor prognosis. But how to identify the high-risk subgroup for those with non-MVI solitary HCC patients should be addressed. Interestingly, the A-G score performed well to identify those patients with intermediate- or high-risk of poor prognosis in terms of RFS and OS. For non-MVI solitary HCC patients, those with both tumor markers concomitantly increasing should be strictly followed up and receive necessary adjuvant therapy. On the other hand, patients with an A-G score of 1 had a higher rate of MVI than that of patients with an A-G score of 0, but lower than that of patients with an A-G score of 2, suggesting a higher A-G score significantly correlated with a higher rate of MVI occurrence. It aids in predicting the presence of MVI preoperatively. Although liver cirrhosis was not a prognostic factor and it was not correlated with the A-G score, in the subgroup analysis we found that the A-G score could effectively predict the prognosis of HCC patients with liver cirrhosis. Liver cirrhosis was related to the incidence of postoperative complications. Identifying patients with liver cirrhosis at a high risk of HCC recurrence was beneficial for planning personal treatments and reducing the incidence of liver dysfunction after surgery.

The presence of HBeAg commonly indicated active viral replication with ongoing inflammatory activity and the potential for sustained liver injury. A previous study has revealed that positive HBeAg was associated with an increased risk for HCC and HCC recurrence after hepatectomy (30, 31). Consistently, we also confirmed it was related to prognosis in solitary HCC patients. Moreover, a high A-G score related to a high rate of positive HBeAg, especially for patients with a high A-G score of 2. It suggested the strong inflammatory activity might promote the malignant behavior of HCC. Many studies to date have shown that pathological factors, such as satellite lesions and tumor differentiation, were determinant prognostic factors for HCC recurrence (32). In the present study, the two pathological factors were confirmed to be related to prognosis among solitary HCC patients. Patients with an A-G score of 1 or 2 had the highest rate of satellite lesions and poor tumor differentiation. Both elevated tumor markers could indicate worse pathological features than either one elevated tumor marker or none. Interestingly, patients with either elevated AFP or GGT had larger tumor diameters than that of those with lower AFP and GGT, smaller than that of patients with both elevated AFP and GGT, indicating the positive correlation between A-G score and tumor size. Moreover, it could further identify a high risk of poor prognosis of solitary HCC patients.

Recently, inflammation-based markers, including NLR and PLR, were identified as prognostic factors for RFS of patients with HCC (33). In the univariate analysis, these indices were significant. Unfortunately, in the Cox model, these indices were not included. The reasons for this discrepancy may be explained by the following two hypotheses: (1) the maximal effect from other factors, such as tumor pathological features and tumor markers; or (2) a specific subgroup of HCC. We further investigated the correlation between NLR/PLR and A-G score. An elevated preoperative NLR and PLR was significant correlated with a high A-G score, especially for PLR. Inflammation-based markers might reflect the tumor malignant potential itself, including the environment around the tumor. It was reasonable that NLR and PLR elevated when the A-G score was high. Taken together, when we classified the BCLC stage of solitary HCC and predicted the prognosis of solitary HCC patients, the A-G score might be taken into account.

The present study has some limitations. First, it was a retrospective and single-center study. It is essential to evaluate its clinical value externally and prospectively. Second, there is no consensus on an optimal cut-off value for AFP or GGT. The cut-off value of AFP was taken from to previous reliable studies. For serum GGT, we adopted a method for determining the optimal pointcut of GGT based on the statistics of maxstat. Based on our classification, the distribution was reasonable (low-risk group:28.9% vs. intermediate-risk group: 50.8% vs. high-risk group: 20.3%). Third, most etiology of underlying liver diseases in this study population was HBV, with a 97.5% of positive HBcAb; whether the results from our study can be applied to HCV-related HCC or multiple HCCs was not certain.

## Conclusions

Preoperative A-G score might be a promising predictor for the prognosis of solitary HCC patients after hepatectomy. This A-G score could effectively and simply identify high-risk cohorts of solitary HCC patients. Meanwhile, it could exactly predict the prognosis of non-MVI solitary HCC patients or patients with liver cirrhosis.

## Data Availability Statement

The raw data supporting the conclusions of this article will be made available by the authors, without undue reservation.

## Author Contributions

LZ proposed the study. GL and JS collected and analyzed the data. GL wrote the first draft. LZ and JS reviewed the manuscript. All authors contributed to the interpretation of the study.

## Conflict of Interest

The authors declare that the research was conducted in the absence of any commercial or financial relationships that could be construed as a potential conflict of interest.

## Supplementary Material

Supplementary Figure 1 Determine the optimal cutoff value for serum GGT using the maximally selected rank statistics. The cutoff value of GGT was 43 U/L. GGT, gamma-glutamyl transferase.

Supplementary Figure 2 The distribution of all solitary HCC patients when stratified by AFP **(A)**, GGT **(B)**, and A-G score **(C)** and key clinicopathological features associated with A-G score **(D)**.
