# Supplementary figures and images for: A-G Score Associated With Outcomes in Solitary Hepatocellular Carcinoma Patients After Hepatectomy
Source: Front Oncol. 2020 Aug 7;10:1286. doi: 10.3389/fonc.2020.01286 (PMC7427538; doi:10.3389/fonc.2020.01286)

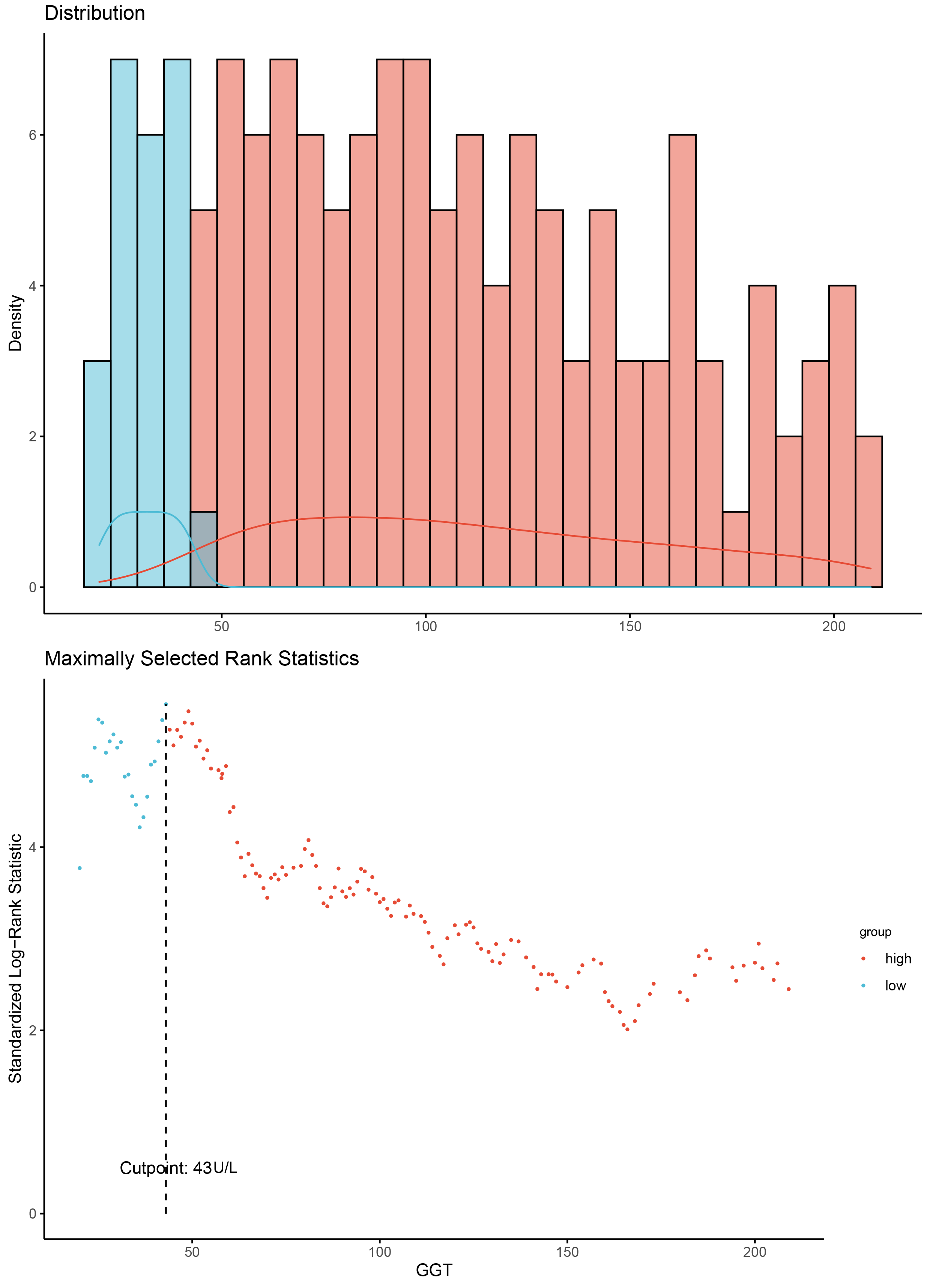

Supplement: Supplementary Figure 1 — Determine the optimal cutoff value for serum GGT using the maximally selected rank statistics. The cutoff value of GGT was 43 U/L. GGT, gamma-glutamyl transferase. [file Image_1.TIF]

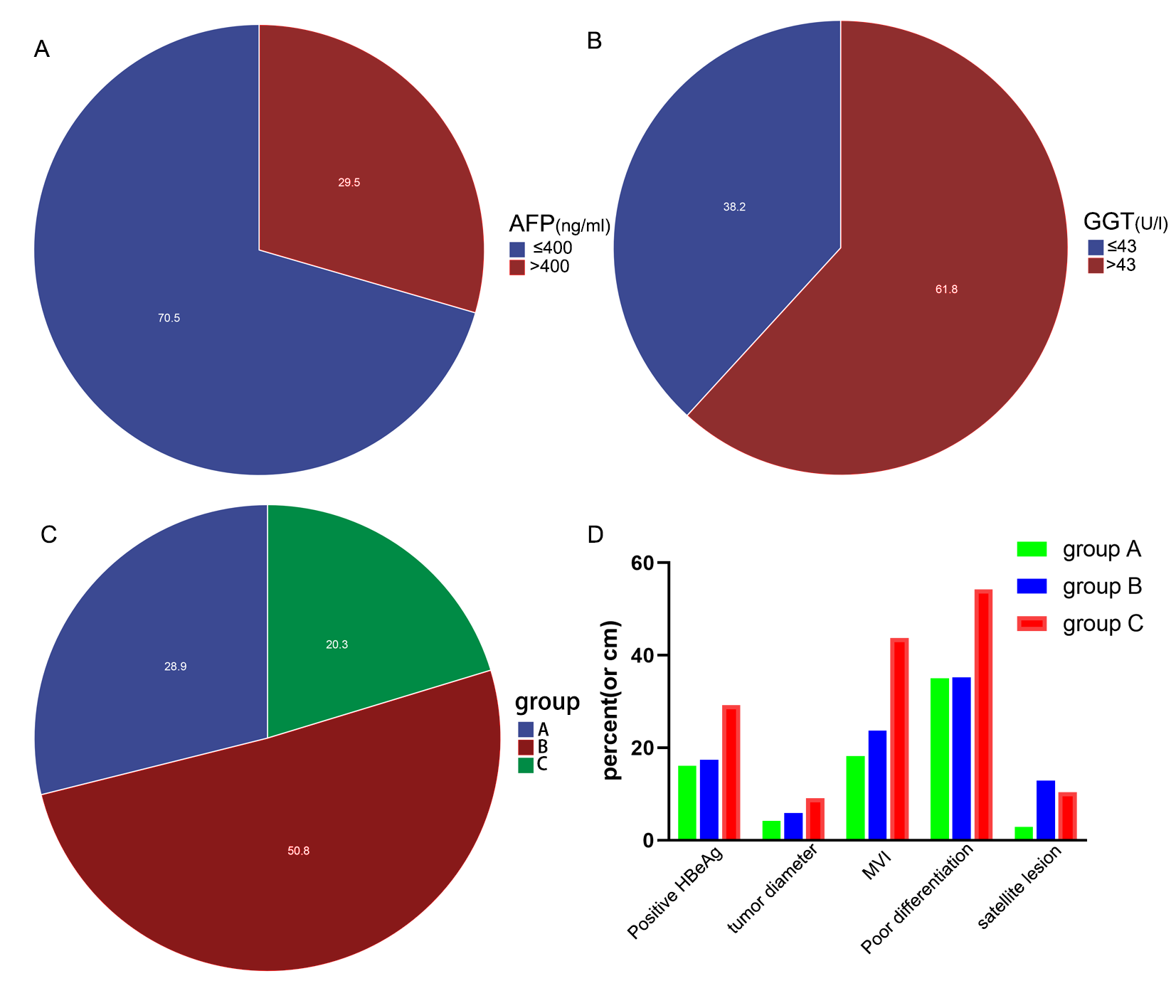

Supplement: Supplementary Figure 2 — The distribution of all solitary HCC patients when stratified by AFP (A), GGT (B), and A-G score (C) and key clinicopathological features associated with A-G score (D). [file Image_2.TIF]
